# Coronal plane femoral bowing in Far East Asians: implications for the strategy of distal femoral resection in total knee arthroplasty

**DOI:** 10.1186/s13018-022-03389-7

**Published:** 2022-11-16

**Authors:** Sang Min Lee, Hak Sang Kim, Jae Hoon Jang, Tae Young Ahn, Jeung Tak Suh, Seung Joon Rhee

**Affiliations:** 1 Department of Orthopaedic Surgery, Research Institute for Convergence of Biomedical Science and Technology, Pusan National University Yangsan Hospital, Yangsan, Korea; 2 Department of Orthopaedic Surgery, Biomedical Research Institute, Pusan National University Hospital, Busan, Korea; 3 Department of Orthopaedic Surgery, Busan Medical Center, Busan, Korea

**Keywords:** Distal femoral resection, Femoral bowing, Intramedullary guide, Total knee arthroplasty, Computer-assisted

## Abstract

**Background:**

The accuracy of distal femoral resection in intramedullary (IM) guided total knee arthroplasty (TKA) depends on femoral morphology and varies according to individual anatomy. This study aimed to characterise coronal plane femoral bowing in Far East Asians according to age, sex, and severity of varus deformity to identify optimal strategies for distal femoral resection in TKA.

**Method:**

Femoral anatomical parameters in 656 patients (M/F = 232:424) were assessed using standing long-leg anteroposterior radiography which was fulfilling strict standard. The femur was divided into three longitudinal segments to measure the segmental anatomical axial deviation from the mechanical axis and intersegmental bowing. Coronal plane femoral bowing pattern was categorised based on combined gross bowing and distal bowing.

**Results:**

Mean hip–knee–ankle angle; neck–shaft angle; proximal, middle, and distal segmental axial differences; mechanical lateral distal femoral angle; and femur length were 6.7 ± 6.8°, 125.0 ± 5.5°, 5.9 ± 1.7°, 6.1 ± 1.1°, 5.3 ± 1.6°, 88.4 ± 2.6°, and 432.3 ± 23.9 mm in male and 8.4 ± 5.5°, 126.4 ± 5.6°, 5.4 ± 1.5°, 6.6 ± 0.9°, 5.6 ± 1.6°, 89.3 ± 2.6°, and 410.6 ± 23.3 mm in female, respectively. Mean proximal, distal, and gross femoral bowing was 0.3 ± 1.8°, − 0.8 ± 1.8°, and − 0.5 ± 2.9° in male and 1.2 ± 1.6°, − 1.0 ± 1.6°, and 0.2 ± 2.7° in female, respectively.

**Conclusions:**

Grossly straight femur with a straight distal part was the most common femoral bowing pattern in Far East Asians. Distal bowing was proved to be a key factor to choose method for distal femoral resection in TKA. Using IM-guide to achieve accurate distal femoral resection in the femora with distal segmental axial deviation between 4–8° and distal bowing less than ± 1° is considered feasible.

## Introduction

Extant evidence indicates that Asians have distinct femoral morphological features compared with Caucasians and other ethnic groups [1, 2]. Among diverse Asian ethnic groups, the Far East Asians including Korean, Chinese, and Japanese are known to be sharing relatively similar femoral anatomical characteristics despite subtle differences in culture and life styles [3–6]. The relationship between femoral anatomy and distal femoral resection has been investigated in Americans, Europeans, and Asians, including Koreans [7–9]. However, to the best of our knowledge, a large-scale analysis of the coronal plane bowing of the femur, mainly in Koreans, is rare.

Distal femoral resection perpendicular to the mechanical femoral axis is essential for realising mechanically aligned total knee arthroplasty (TKA). Conventionally, the procedure is performed using an intramedullary (IM) rod and attachable resection guide with preset valgus angle (IM-guide). However, the accuracy of the resection technique using the IM-guide is largely dependent on femoral morphology and varies widely according to individual anatomy [10, 11]. Despite recent developments in computer-assisted surgery and robotic surgery that have improved accuracy and reproducibility, these tools are not easily accessible in all clinics or countries [12, 13]. The initial cost of purchasing these devices is high, and insurance systems currently do not fully support the use of computer-assisted or robotic devices in some countries. Nevertheless, achieving accurate distal femoral resection according to surgical plan remains a key procedure in TKA, and delineating the coronal plane femoral bowing of Far East Asians will be a useful guide.

This study hypothesised that (1) coronal plane femoral bowing in different demographic groups in Far East Asians will differ significantly. (2) Grossly laterally bowed femur will be revealed as a dominant bowing pattern in Far East Asian femora. (3) Coronal plane bowing of the distal femur will affect the feasibility of IM-guided distal femoral resection in TKA.

## Patients and methods

This study was approved by the institutional review board of our hospital (2106-021-104). Informed consent was obtained from all patients. From January 2015 to January 2021, 952 primary TKAs were performed in our institution. We reviewed preoperative long-leg standing anteroposterior radiography (orthoroentgenogram) of the operated patients. In total, 656 images which fulfilled strict radiographic standards were included. All orthoroentgenograms were obtained with the patient standing and patellae facing forward to place the lower limb in neutral rotation. The radiographic beam was centred at the knee joint line level. The radiographic position of the patella and exposed lesser trochanteric area upon the femoral medial cortex were used to determine the adequacy of radiography. Femora with posttraumatic or congenital deformities were excluded.

### Measurements

**Fig. 1:**
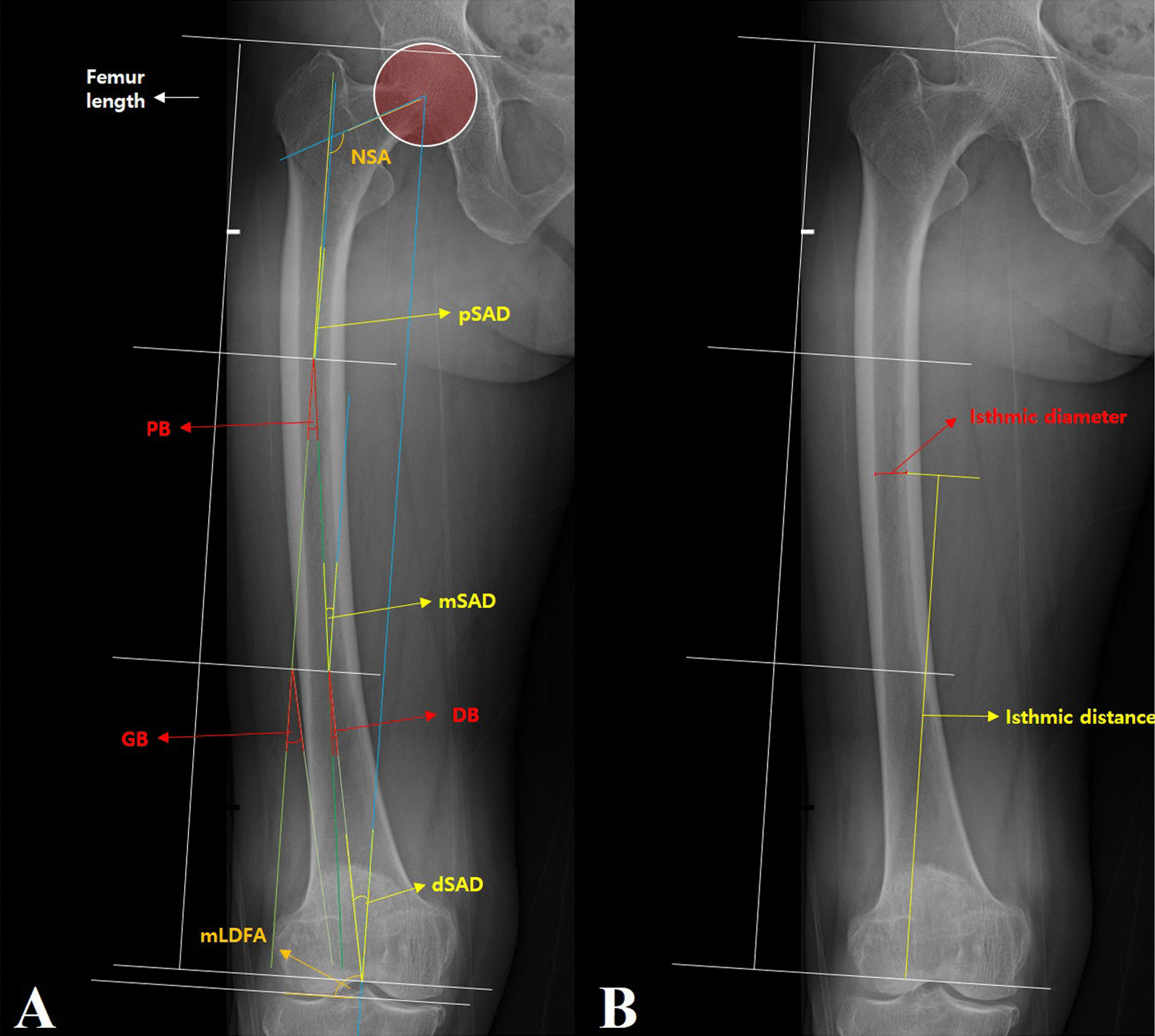
**A** Segmental anatomical axial deviation from the femora mechanical axis was measured for each of the three sections and intersegmental bending was calculated. Note that bending angles are not the mirrored angles of SAD. **B** Isthmic distance from the top of the intercondylar notch and isthmic width in the femoral intramedullary canal was measured. *NSA* neck–shaft angle, *pSAD* proximal segmental axial difference, PB proximal femoral bowing, *mSAD* middle segmental axial difference, *GB* gross femoral bowing, *DB* distal femoral bowing, *dSAD* distal segmental axial difference, *mLDFA* mechanical lateral distal femoral angle

In the included orthoroentgenograms, hip–knee–ankle (HKA) angle was measured, and the femur was separated into three serial sections for analysis. Whole femoral length was defined as the length from the greater trochanteric tip to top of the intercondylar notch [14] and was divided into proximal, middle, and distal segments of equal length. For each segment, the angle between the anatomical axis of the femoral segment and mechanical femoral axis was measured and was defined as the proximal, middle, and distal segmental axial difference (pSAD, mSAD, and dSAD, respectively). The angles between the anatomical axis of the femoral neck and proximal segment (neck–shaft angle [NSA]) and between the mechanical femoral axis and coronal femoral joint line (mechanical lateral distal femoral angle [mLDFA]) were also measured (Fig. 1). The relative angles between the femoral proximal and middle segments (proximal femoral bowing [PB]), middle and distal segments (distal femoral bowing [DB]), and proximal and distal segments (gross femoral bowing [GB]) were calculated by subtracting the SAD of a relatively proximal segment from that of a relatively distal segment. Positive values > 1° were considered lateral bowing, while negative values >  − 1° were considered medial bowing in defining PB and DB. For GB, ± 2° was set as the cut-off value of defining lateral and medial bowing. Femoral bowing was categorised into nine patterns according to the combination of GB and DB. The ratio of straight DB to serial dSAD was calculated. Isthmic distance from the top of the intercondylar notch and isthmic width in the femoral intramedullary canal were measured. The isthmic portion location was defined as the ratio of the length from the top of the intercondylar notch to the isthmus to whole femoral length. All measurements were repeated twice by three observers at a 6-week interval in 30 randomly selected patients to evaluate intra- and interobserver reliability. The intraclass correlation coefficients (ICCs) of intra-observer and interobserver reliability for all measurements were 0.958 (95% CI 0.918–0.979, *p* < 0.001) and 0.843 (95% CI 0.452–0.933, *p* < 0.001), respectively. As good reproducibility of the measurements was verified, measurements by one of the researchers (blinded for review) were used for all subsequent analyses. Femur models reflecting the measured parameters were rendered using PowerPoint 2016 software (Microsoft) and Photoshop (Adobe).

### Statistical analysis

Statistical analyses were performed using SPSS (version 21.0, SPSS, Chicago, IL). Angular measurements are reported as the mean ± SD and range. All data were rounded off to two decimal places. To compare between two groups, an independent *t* test or Mann–Whitney U test was used for numerical variables, and Pearson’s chi-squared test or Fisher’s exact test for categorical variables. Correlations of GB with PB and DB were assessed using Spearman’s correlation coefficient. Statistical significance was set at *p* < 0.05.

## Results

The mean values of HKA, NSA, pSAD, mSAD, dSAD, mLDFA, and femur length were 6.7 ± 6.8°, 125.0 ± 5.5°, 5.9 ± 1.7°, 6.1 ± 1.1°, 5.3 ± 1.6°, 88.4 ± 2.6°, and 432.3 ± 23.9 mm in male and 8.4 ± 5.5°, 126.4 ± 5.6°, 5.4 ± 1.5°, 6.6 ± 0.9°, 5.6 ± 1.6°, 89.3 ± 2.6°, and 410.6 ± 23.3 mm in female, respectively. HKA (*p* = 0.002), NSA (*p* = 0.002), mSAD (*p* < 0.001), dSAD (*p* = 0.027), and mLDFA (*p* < 0.001) were significantly larger in female, whereas pSAD (*p* = 0.004) was larger in male. The mean values of PB, DB, and GB were 0.3 ± 1.8°, − 0.8 ± 1.8°, and − 0.5 ± 2.9° in male and 1.2 ± 1.6°, –1.0 ± 1.6°, and 0.2 ± 2.7° in female, respectively. PB (*p* < 0.001) and GB (*p* = 0.002) were significantly larger in female. Mean distance, diameter, and location of the femoral isthmic portion were 245.0 ± 23.8 mm, 12.2 ± 1.9 mm, and 56.6 ± 4.0% in male and 232.1 ± 23.1 mm, 11.8 ± 2.1 mm, and 56.5 ± 5.7% in female, respectively. Compared to female, male had longer (*p* < 0.001) femora with longer isthmic distance (*p* < 0.001) from the intercondylar notch. However, the isthmic diameter and location in the whole femur were similar between male and female (Table 1).

**Table 1:** Comparison of the parameters according to the sex difference

	Male (*n* = 232)	Female (*n* = 424)	*p* value
Age (years old)	69.4 ± 8.2 (50 to 89)	70.5 ± 6.8 (52 to 89)	
Hip–knee–ankle angle (HKA, °)	6.7 ± 6.8 (− 19.6 to 27.6)	8.4 ± 5.5 (− 13.4 to 25.4)	0.002*
Neck–shaft angle (NSA, °)	125.0 ± 5.5 (109.9 to 137.2)	126.4 ± 5.6 (110.6 to 142.7)	0.002*
pSAD (°)	5.9 ± 1.7 (1.1 to 12.2)	5.4 ± 1.5 (1.0 to 10.4)	0.004*
mSAD (°)	6.1 ± 1.1 (3.5 to 8.8)	6.6 ± 0.9 (4.1 to 8.8)	< 0.001*
dSAD (°)	5.3 ± 1.6 (2.1 to 10.3)	5.6 ± 1.6 (1.8 to 11.3)	0.027*
PB (mSAD–pSAD, °)	0.3 ± 1.8 (− 6.3 to 5.2)	1.2 ± 1.6 (− 3.7 to 7.7)	< 0.001*
DB (dSAD–mSAD, °)	− 0.8 ± 1.8 (− 6.0 to 5.8)	− 1.0 ± 1.6 (− 5.7 to 5.3)	0.229
GB (dSAD–pSAD, °)	− 0.5 ± 2.9 (− 7.7 to 9.1)	0.2 ± 2.7 (− 6.7 to 9.8)	0.002*
mLDFA (°)	88.4 ± 2.6 (80.7 to 96.7)	89.3 ± 2.6 (81 to 98)	< 0.001*
Femur length (mm)	432.3 ± 23.9 (365.1 to 517.1)	410.6 ± 23.3 (342 to 481.5)	< 0.001*
Isthmic distance (mm)	245.0 ± 23.8 (136.7 to 321.7)	232.1 ± 23.1 (121.1 to 297.0)	< 0.001*
Isthmic location (%)	56.6 ± 4.0 (34.1 to 68.7)	56.5 ± 5.7 (40.2 to 74.0)	0.844
Isthmic diameter (mm)	12.2 ± 1.9 (8.6 to 19.1)	11.8 ± 2.1 (7.0 to 16.9)	0.076

*pSAD* proximal segmental axial deviation, *mSAD* middle segmental axial deviation, *dSAD* distal segmental axial deviation, *PB* proximal bowing, *DB* distal bowing, *GB* gross bowing, *mLDFA* mechanical lateral distal femoral angle

*p < 0.05

Comparison of age groups younger (*n* = 114) and older (*n* = 118) than 70 years of age revealed that HKA angle (*p* = 0.033) and mLDFA (*p* = 0.028) were significantly different according to age groups in male. HKA angle and mLDFA were 5.6 ± 7.3° and 88.0 ± 2.8°, respectively, in the younger group, and 7.8 ± 6.0° and 88.8 ± 2.4°, respectively, in the older group. In female, PB (*p* = 0.040) and GB (*p* = 0.021) were significantly larger in the older group (*n* = 235; 1.3 ± 1.7° and 0.4 ± 2.9°, respectively) than in the younger group (*n* = 189; 1.0 ± 1.5° and − 0.1 ± 2.4°, respectively). These data suggested that the tendency and degree of femoral bowing were similar regardless of age in male, whereas the degree of PB and GB increased with age in female (Tables 2 and 3).

**Table 2:** Comparison of the parameters according to the age group in male

Male	50–69 years (*n* = 114)	70–89 years (*n* = 118)	*p* value
Hip–knee–ankle angle (HKA, °)	5.6 ± 7.3 (− 19.6 to 24.5)	7.8 ± 6.0 (− 11.6 to 27.6)	0.033*
Neck–shaft angle (NSA, °)	125.2 ± 5.4 (109.9 to 137.1)	124.8 ± 5.7 (112.7 to 137.2)	0.583
pSAD (°)	6.0 ± 1.8 (1.1 to 12.2)	5.7 ± 1.6 (1.2 to 10.3)	0.210
mSAD (°)	6.1 ± 1.1 (3.8 to 8.8)	6.1 ± 1.1 (3.5 to 8.8)	0.954
dSAD (°)	5.2 ± 1.7 (2.1 to 10.3)	5.4 ± 1.6 (2.1 to 9.5)	0.349
PB (mSAD–pSAD, °)	0.1 ± 1.9 (− 6.3 to 4.6)	0.4 ± 1.7 (− 4.5 to 5.2)	0.212
DB (dSAD–mSAD, °)	− 0.9 ± 1.9 (− 4.9 to 5.8)	− 0.7 ± 1.7 (− 6.0 to 3.2)	0.370
GB (dSAD–pSAD, °)	− 0.8 ± 2.9 (− 7.7 to 9.1)	− 0.3 ± 2.8 (− 6.7 to 6.6)	0.180
mLDFA (°)	88.0 ± 2.8 (80.7 to 96.7)	88.8 ± 2.4 (82.6 to 96.7)	0.028*
Femur length (mm)	433.9 ± 24.3 (368.9 to 501.5)	430.8 ± 23.6 (365.1 to 517.1)	0.335
Isthmic distance (mm)	246.5 ± 22.1 (207.1 to 309.0)	243.5 ± 25.4 (136.7 to 321.7)	0.323
Isthmic location (%)	56.7 ± 3.4 (48.9 to 66.7)	56.6 ± 4.6 (34.1 to 68.7)	0.975
Isthmic diameter (mm)	12.2 ± 2.0 (8.6 to 18.3)	12.2 ± 1.9 (8.8 to 19.1)	0.787

*pSAD* proximal segmental axial deviation, *mSAD* middle segmental axial deviation, *dSAD* distal segmental axial deviation, *PB* proximal bowing, DB distal bowing, *GB* gross bowing, *mLDFA* mechanical lateral distal femoral angle

*p < 0.05

**Table 3:** Comparison of the parameters according to the age group in female

Female	50–69 years (*n* = 189)	70–89 years (*n* = 235)	*p* value
Hip–knee–ankle angle (HKA, °)	8.6 ± 4.9 (− 7.5 to 24.5)	8.3 ± 5.9 (− 13.4 to 25.4)	0.914
Neck–shaft angle (NSA, °)	126.9 ± 5.6 (111.6 to 141.7)	126.0 ± 5.6 (110.6 to 142.7)	0.112
pSAD (°)	5.6 ± 1.3 (1.1 to 8.6)	5.3 ± 1.6 (1.0 to 10.4)	0.073
mSAD (°)	6.6 ± 0.8 (4.1 to 8.8)	6.6 ± 0.9 (4.1 to 8.7)	0.475
dSAD (°)	5.5 ± 1.5 (2.3 to 10.7)	5.8 ± 1.7 (1.8 to 11.3)	0.065
PB (mSAD–pSAD, °)	1.0 ± 1.5 (− 2.7 to 7.7)	1.3 ± 1.7 (− 3.7 to 6.7)	0.040*
DB (dSAD–mSAD, °)	− 1.1 ± 1.5 (− 4.2 to 4.1)	− 0.9 ± 1.8 (− 5.7 to 5.3)	0.204
GB (dSAD–pSAD, °)	− 0.1 ± 2.4 (− 5.4 to 9.6)	0.4 ± 2.9 (− 6.7 to 9.8)	0.021*
mLDFA (°)	89.2 ± 2.5 (81.6 to 95.3)	89.3 ± 2.7 (81.0 to 98.0)	0.728
Femur length (mm)	411.8 ± 22.0 (363.0 to 481.5)	409.7 ± 24.3 (342.0 to 472.5)	0.392
Isthmic distance (mm)	232.9 ± 21.8 (164.0 to 287.5)	231.5 ± 24.1 (121.1 to 297.0)	0.849
Isthmic location (%)	56.7 ± 5.5 (40.2 to 73.3)	56.4 ± 5.8 (40.8 to 74.0)	0.753
Isthmic diameter (mm)	11.8 ± 2.1 (7.3 to 10.7)	11.8 ± 2.1 (7.0 to 16.7)	0.781

*pSAD* proximal segmental axial deviation, *mSAD* middle segmental axial deviation, *dSAD* distal segmental axial deviation, *PB* proximal bowing, *DB* distal bowing, *GB* gross bowing, *mLDFA* mechanical lateral distal femoral angle

*p < 0.05

In male, mean HKA was 5.9 ± 2.8° and 13.5 ± 3.6° in the under (*n* = 135) and over (*n* = 70) 10° groups, respectively. dSAD (*p* < 0.001), DB (*p* = 0.007), GB (*p* = 0.007), and mLDFA (*p* < 0.001) were significantly larger in male with HKA > 10°. In female, mean HKA was 6.4 ± 2.4° and 13.4 ± 2.8° in the under (*n* = 236) and over (*n* = 162) 10° groups, respectively. All the angular values except for NSA and mSAD were significantly larger in the HKA > 10° group than in the HKA < 10° group in female (Tables 4 and 5).

**Table 4:** Comparison of the parameters according to the HKA angle in male

Male	HKA 0–10° (*n* = 135)	HKA 10° < (*n* = 70)	*p* value
Age (years old)	70.0 ± 8.0 (52.0 to 88.0)	69.8 ± 7.8 (53.0 to 89.0)	
Hip–knee–ankle angle (HKA, °)	5.9 ± 2.8 (0.1 to 10.0)	13.5 ± 3.6 (10.1 to 27.6)	< 0.001*
Neck–shaft angle (NSA, °)	125.1 ± 5.4 (112.3 to 137.2)	124.1 ± 5.4 (109.9 to 133.9)	0.212
pSAD (°)	6.0 ± 1.7 (1.8 to 12.2)	5.7 ± 1.7 (1.1 to 10.3)	0.442
mSAD (°)	6.1 ± 1.0 (3.8 to 8.5)	6.2 ± 1.1 (3.5 to 8.8)	0.556
dSAD (°)	5.1 ± 1.5 (2.1 to 9.5)	6.0 ± 1.6 (3.0 to 10.3)	< 0.001*
PB (mSAD–pSAD, °)	0.1 ± 1.8 (− 6.3 to 5.2)	0.6 ± 1.6 (− 4.0 to 4.6)	0.102
DB (dSAD–mSAD, °)	− 1.0 ± 1.6 (− 4.8 to 3.4)	− 0.3 ± 2.0 (− 4.9 to 5.8)	0.007*
GB (dSAD–pSAD, °)	− 0.8 ± 2.7 (− 7.7 to 6.6)	0.3 ± 2.9 (− 5.9 to 9.1)	0.007*
mLDFA (°)	88.3 ± 2.1 (82.6 to 95.4)	89.9 ± 2.3 (85.2 to 96.7)	< 0.001*
Femur length (mm)	433.8 ± 25.9 (368.9 to 517.1)	429.5 ± 21.5 (365.1 to 483.0)	0.335
Isthmic distance (mm)	245.7 ± 26.4 (136.7 to 321.7)	243.0 ± 19.2 (197.2 to 286.2)	0.726
Isthmic location (%)	56.6 ± 4.5 (34.1 to 68.7)	56.6 ± 3.1 (48.1 to 63.6)	0.565
Isthmic diameter (mm)	12.3 ± 2.0 (8.6 to 18.3)	12.0 ± 1.8 (8.7 to 16.8)	0.413

*pSAD* proximal segmental axial deviation, *mSAD* middle segmental axial deviation, *dSAD* distal segmental axial deviation, *PB* proximal bowing, *DB* distal bowing, *GB* gross bowing, *mLDFA* mechanical lateral distal femoral angle

*p < 0.05

**Table 5:** Comparison of the parameters according to the HKA angle in female

Female	HKA 0–10° (*n* = 236)	HKA 10° < (*n* = 162)	*p* value
Age (years old)	70.5 ± 6.8 (52.0 to 89.0)	70.7 ± 6.6 (55.0 to 86.0)	
Hip–knee–ankle angle (HKA, °)	6.4 ± 2.4 (0.1 to 10.0)	13.4 ± 2.8 (10.1 to 25.4)	< 0.001*
Neck–shaft angle (NSA, °)	126.4 ± 5.6 (111.7 to 141.7)	126.5 ± 5.9 (110.6 to 142.7)	0.865
pSAD (°)	5.7 ± 1.4 (1.0 to 10.4)	4.9 ± 1.5 (1.0 to 7.6)	< 0.001*
mSAD (°)	6.6 ± 0.9 (4.1 to 8.8)	6.6 ± 0.8 (4.1 to 8.8)	0.440
dSAD (°)	5.2 ± 1.4 (2.0 to 10.5)	6.3 ± 1.5 (3.5 to 11.3)	< 0.001*
PB (mSAD–pSAD, °)	0.9 ± 1.5 (− 3.7 to 6.5)	2.0 ± 1.6 (− 1.7 to 7.7)	< 0.001*
DB (dSAD–mSAD, °)	− 1.4 ± 1.4 (− 5.0 to 3.9)	− 0.1 ± 1.7 (− 4.0 to 5.3)	< 0.001*
GB (dSAD–pSAD, °)	− 0.5 ± 2.4 (− 6.7 to 9.5)	1.5 ± 2.8 (− 3.5 to 9.8)	< 0.001*
mLDFA (°)	88.7 ± 2.3 (82.5 to 96.3)	90.9 ± 2.1 (85.9 to 98.0)	< 0.001*
Femur length (mm)	409.9 ± 23.6 (342.0 to 481.5)	411.6 ± 23.0 (360.0 to 469.8)	0.528
Isthmic distance (mm)	232.8 ± 20.6 (164.0 to 297.0)	230.4 ± 25.7 (121.1 to 287.5)	0.265
Isthmic location (%)	59.8 ± 5.4 (40.2 to 74.0)	58.0 ± 5.0 (41.9 to 70.2)	0.200
Isthmic diameter (mm)	11.8 ± 2.0 (7.0 to 10.7)	11.9 ± 2.1 (7.3 to 16.8)	0.614

*pSAD* proximal segmental axial deviation, *mSAD* middle segmental axial deviation, *dSAD* distal segmental axial deviation, *PB* proximal bowing, *DB* distal bowing, *GB* gross bowing, *mLDFA* mechanical lateral distal femoral angle

*p < 0.05

**Fig. 2:**
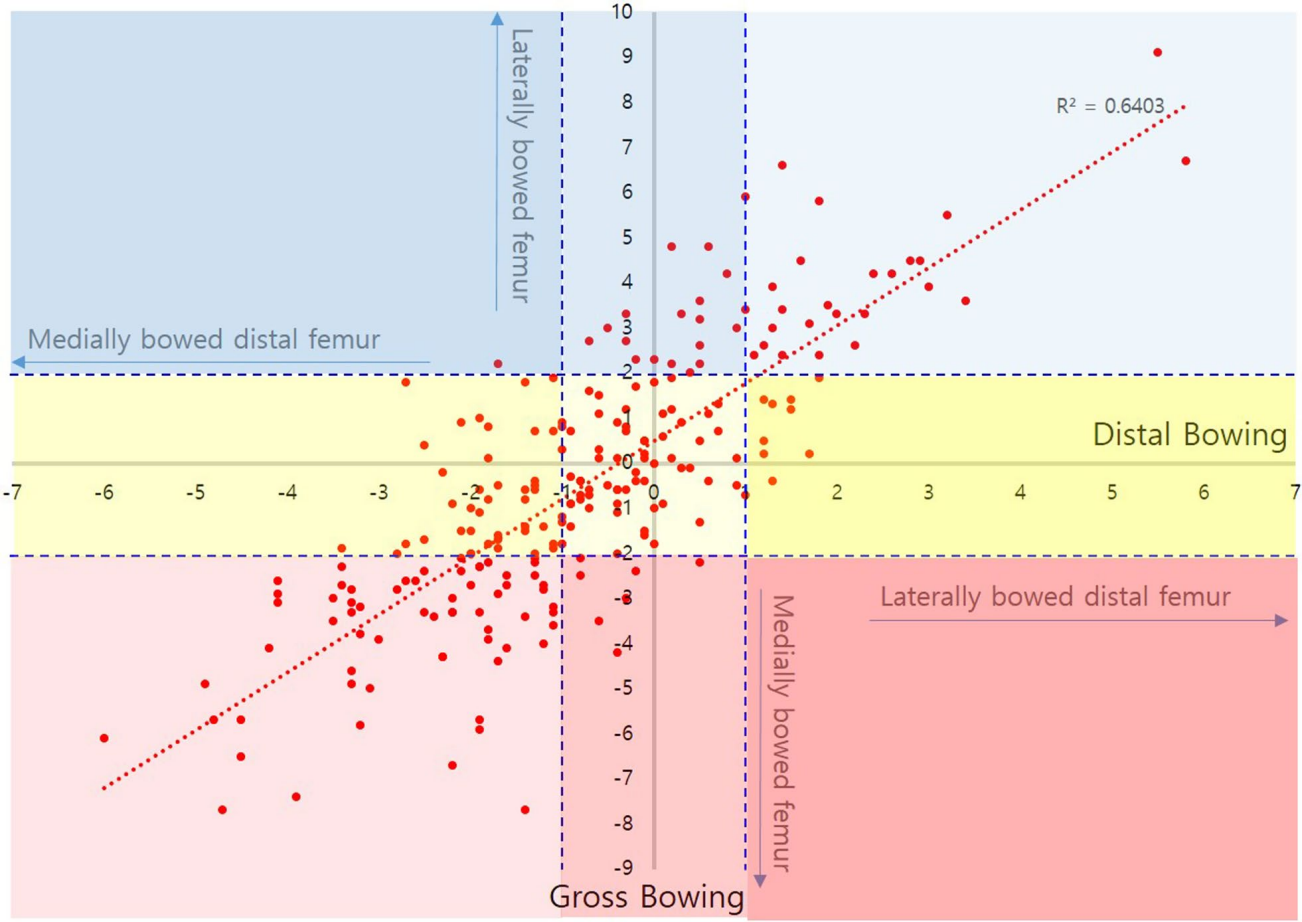
Correlation between distal femoral bowing and gross bowing in male

**Fig. 3:**
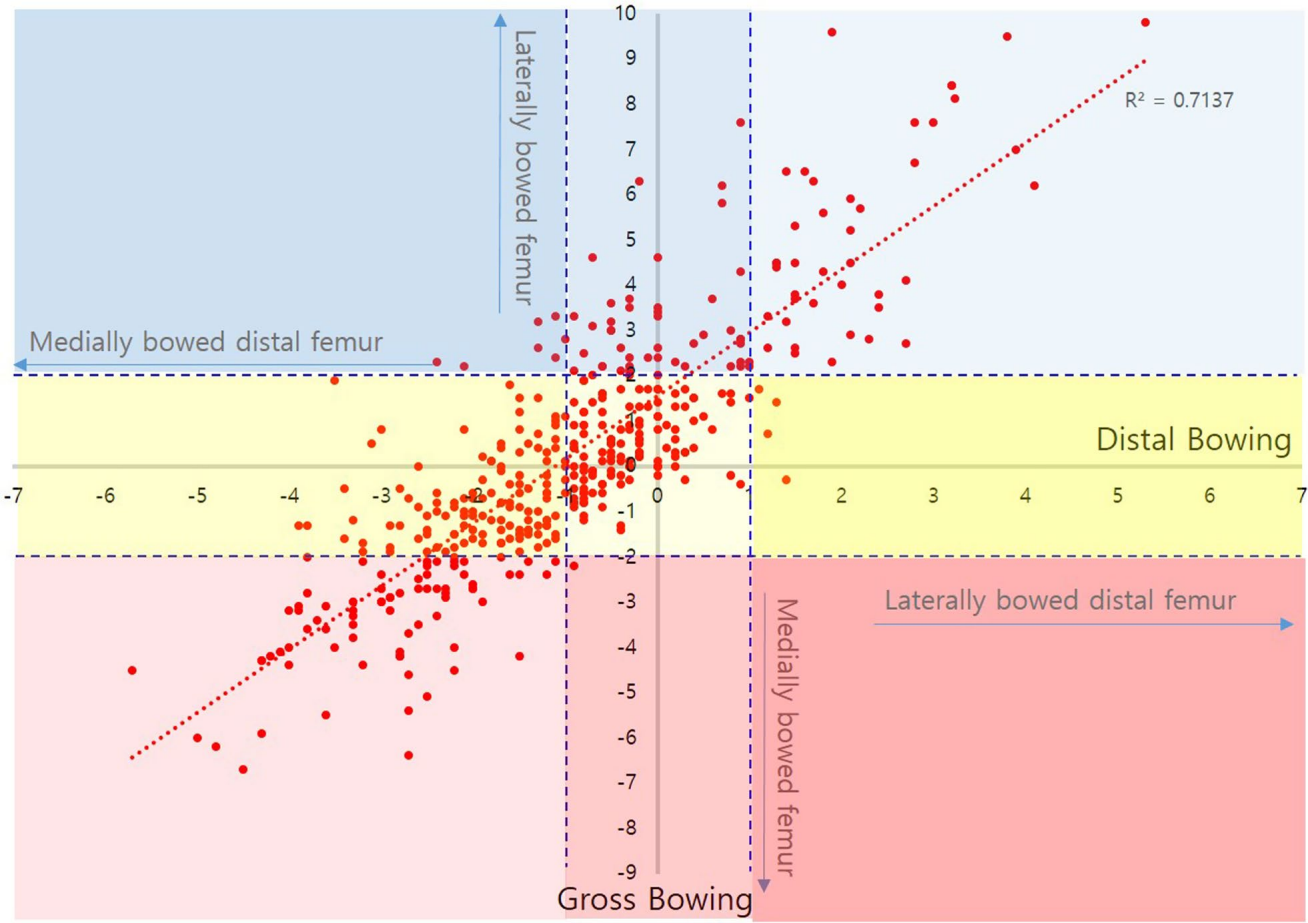
Correlation between distal femoral bowing and gross bowing in female

Correlation coefficients of GB with PB and DB were 0.802 (*p* < 0.05) and 0.800 (*p* < 0.05), respectively, in male and 0.836 (*p* < 0.05) and 0.845 (*p* < 0.05), respectively, in female. Both PB and DB were strongly positively correlated with GB, with a similar strength of correlation (Figs. 2 and 3).

**Fig. 4:**
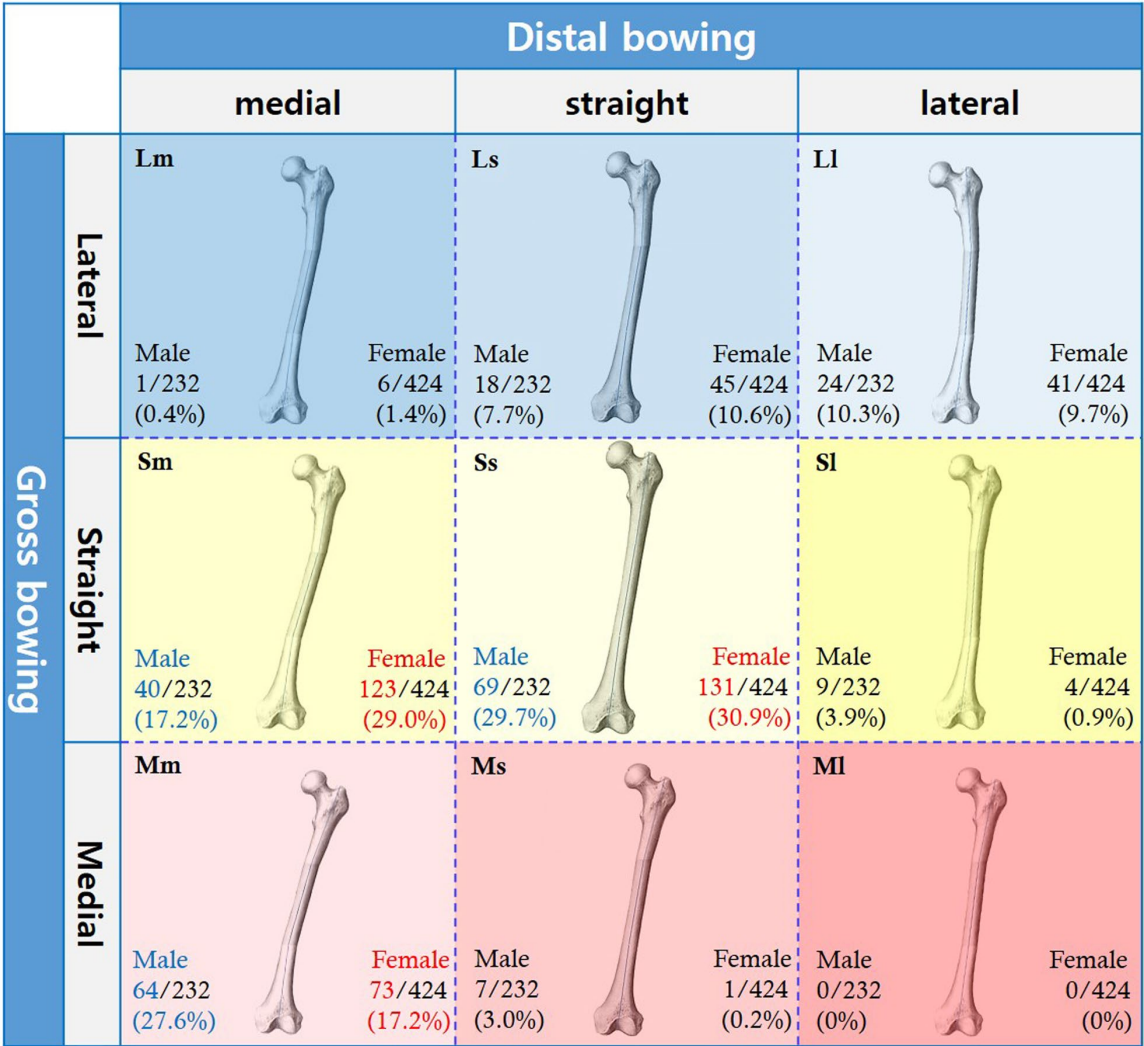
Types of coronal plane femoral bowing and their distribution. *Lm* grossly laterally bowed femora with medially bowed distal part, *Ls* grossly laterally bowed femora with straight distal part, *Ll* grossly laterally bowed femora with laterally bowed distal part, *Sm* grossly straight femur with medially bowed distal part, *Ss* grossly straight femur with straight distal part, *Sl* grossly straight femur with laterally bowed distal part, *Mm* grossly medially bowed femur with medially bowed distal part, *Ms* grossly medially bowed femur with straight distal part, *Ml* grossly medially bowed femur with laterally bowed distal part

A grossly straight femur with straight distal part (Ss) was the most common pattern in both male (*n* = 69, 29.7%) and female (*n* = 131, 30.9%). A grossly straight femur with medially bowed distal part (Sm) was the second most common pattern in female (*n* = 123, 29.0%) and the third most common pattern in male (*n* = 40, 17.2%). A grossly medially bowed femur with medially bowed distal part (Mm) was the second most common pattern in male (*n* = 64, 27.6%) and the third most common pattern in female (*n* = 73, 17.2%). Nearly 10% of the population (24 male and 41 female) had a grossly laterally bowed femur with laterally bowed distal part (Ll), and a slightly smaller proportion of the population (18 male/45 female) had a grossly laterally bowed femur with straight distal part (Ls). Less than 5% of the population had the remaining four patterns. In particular, none of the 656 participants had a grossly medially bowed femur with laterally bowed distal part (Fig. 4).

**Fig. 5:**
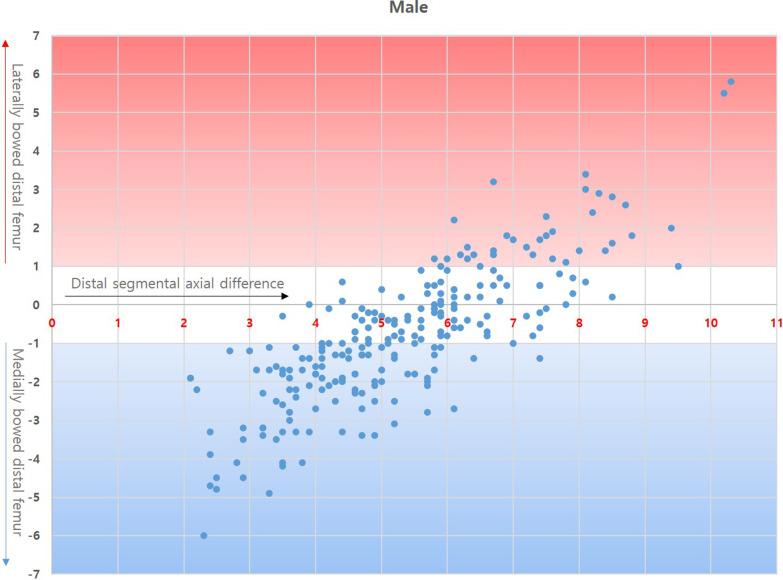
Correlation between distal segmental axial difference and distal femoral bowing in male

**Fig. 6:**
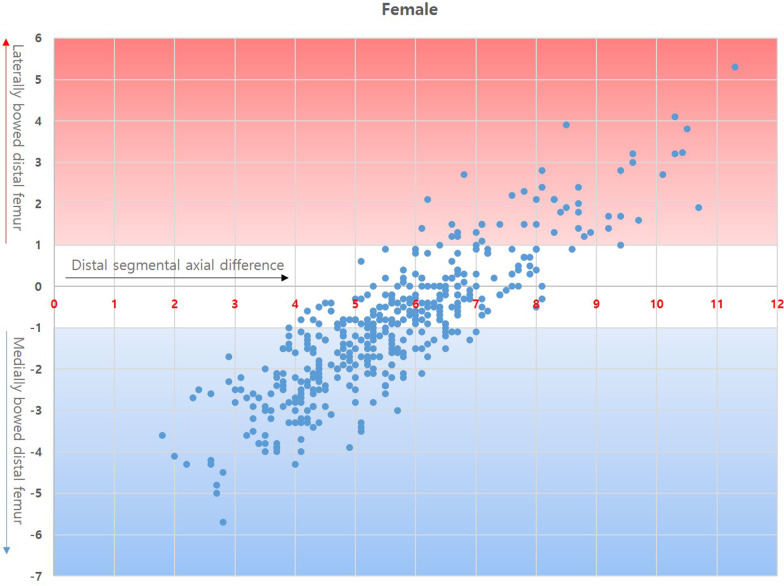
Correlation between distal segmental axial difference and distal femoral bowing in female

DB pattern analysis according to the dSAD revealed that straight DB was the dominant DB pattern observed in the femora with 5–7.9° dSAD, whereas in femora with dSAD < 4° or > 8°, laterally or medially bowed DB prevailed (Table 6, Figs. 5 and 6).

**Table 6:** Distal bowing pattern according to the distal segmental axial deviation (dSAD)

DB	dSAD							
	2°–2.9° (*n* = 14)	3°–3.9° (*n* = 35)	4°–4.9° (*n* = 56)	5°–5.9° (*n* = 54)	6°–6.9° (*n* = 35)	7°–7.9° (*n* = 22)	8° < (*n* = 16)	Total (*n* = 232)
**Male**								
Medial	14	33	38	17	2	1	0	105
Straight	0	2	18	36	23	12	3	94
Lateral	0	0	0	1	10	9	13	33
Mean DB	− 3.6	− 2.4	− 1.5	− 0.7	0.4	0.6	2.4	− 0.8
% Straight	0	5.7%	32.1%	66.7%	65.7%	54.5%	18.8%	40.5%

DB	dSAD							
	2°–2.9° (*n* = 14)	3°–3.9° (*n* = 43)	4°–4.9° (*n* = 92)	5°–5.9° (*n* = 112)	6°–6.9° (*n* = 90)	7°–7.9° (*n* = 35)	8° < (*n* = 38)	Total (*n* = 424)
**Female**								
Medial	14	42	78	54	13	1	0	202
Straight	0	1	14	58	70	26	8	177
Lateral	0	0	0	0	7	8	30	45
Mean DB	− 3.7	− 2.7	− 2.0	− 1.2	− 0.2	0.5	2.0	− 1.0
% Straight	0	2.3%	15.2%	51.8%	77.8%	74.3%	21.1%	41.7%

*DB* distal bowing, *% straight* ratio of straight distal bowing in the given dSAD

## Discussion

Our first hypothesis was that coronal plane femoral bowing in different demographic groups in Far East Asians would differ, and the hypothesis was proven to be true. Significant differences were observed between male and female in most parametric values, except for distal bowing and isthmic location. In female, bowing factors including the mSAD, dSAD, PB, and GB contributed to a more laterally bowed femur, whereas the NSA, pSAD, and mLDFA contributed to a more medially bowed femur compared to that in male. However, rendered images of the femur with application of the mean measurement value did not reveal gross differences between male and female, with the exception of size factors. This is because the parametric differences were quantitatively subtle despite being statistically significant. In the comparative analysis of age groups, male had a similar tendency and degree of femoral bowing regardless of age. On the other hand, the degree of PB and GB was significantly higher in the older female group than in the younger female group. These results may suggest that progressive femoral deformation according to ageing predominantly affects the proximal part of the femur in female. Notably, subgrouping by age of 70 was performed because total knee arthroplasty has been commonly indicated in patients who are older than 60 years in authors’ country by national insurance issue. So, the patient population was mainly comprised of age between 50 and 90 at most that we decided to divide the patients in two groups around the midpoint between that age ranges. The trends of differences in size factors were predictable based on common knowledge, while the exact scale is comparable to reports in the literature [6, 15–17]. Several studies have examined the femoral coronal plane anatomy in Far East Asians. Mochizuki et al. [18] reported higher femoral lateral bowing in female. Our comparison of male and female revealed that GB was significantly higher (*p* = 0.002) in female than in male, similar to the findings of Mochizuki et al. Mean GB was − 0.5 in male and 0.2 in female, which were both interpreted as straight GB according to our categorisation despite the statistically significant difference. Abdelaal et al. [3] reported a ‘parenthesis’ shape of femur bowing in Japanese individuals based on mathematical calculation of the radius of curvature using computerised femur models. They reported that femur bowing differed between the Japanese and Chinese, which were reported by Tang et al. to have a ‘J’ shape [17]. Premising a laterally bowed femoral curve may have resulted in erroneous recognition of coronal femoral shape in Tang et al.’s report. In our study, we confirmed a notable ratio of medially bowed femoral shapes which could not be interpreted with the radii of medially centred circles. Such phenotypic differences between Far East Asians, if they do exist, may be due to the influence of accumulated differences in lifestyle and genetic factors. For instance, the Chinese tend to sit on chairs, whereas the Koreans and Japanese tend to sit on the floor; these habits have been practiced for centuries. Moreover, when sitting on the floor, the Koreans prefer adopting a cross-legged position, while the Japanese prefer kneeling. Further research investigating the influence of ethnic cultural factors on variations in femoral coronal morphology is warranted.

Regarding the second hypothesis, high prevalence of laterally bowed femora has been documented in Far East Asians, especially Koreans [3, 5, 7, 19–21]. In the literature, the term ‘bowing’ was generally used to describe the diverging femoral mechanical–anatomical axis in the distal femoral section, with less consideration of other sections. We classified femoral bowing as distal, proximal, and gross ones by defining the femur as a longitudinal tri-sectional structure, in contrast with studies that regarded the femur as a bi-sectional structure [22, 23]. In bi-sectional anatomy, most ‘bowed’ femora naturally have a grossly laterally bowed shape, whereas the bowing pattern in tri-sectional anatomy is more complex. In this study, Ss was the most common femoral bowing pattern, comprising approximately one-third of the entire study population regardless of sex (Fig. 4). Notably, patterns with gross lateral bowing (18.4% in male and 21.7% in female) or distal lateral bowing (14.2% in male and 10.6% in female) did not constitute more than 20% of the entire study population, although these patterns are more commonly reported. Mm was the second most common pattern in male and the third most common pattern in female. Moreover, a medially bowed distal femur, conventionally referred to as ‘reversely bowed distal femur’, was observed in 45.2% of male and 47.6% of female. According to our results, the representative femoral shape in Koreans was almost straight. So, grossly laterally bowed femur was revealed as a minor bowing pattern in Far East Asian femora in the present study (Fig. 4). Considering that description of the femoral anatomy has been often communicated depending on this tri-segmental system as we often see in fracture treatment, it was regarded reasonable approach to interpret the coronal plane femoral anatomy in this fashion though it is sometimes impossible or not reproducible in severely deformed femur.

Thirdly, we hypothesised that coronal plane bowing of the distal femur would affect the feasibility of IM-guided distal femoral resection in TKA. Regarding the hypothesis, the DB pattern is particularly important because the isthmic portion of the femoral intramedullary canal is predominantly located in the middle segment of the femur. In this study, the ratio of the isthmic location to whole femur was approximately 56% from the top of the intercondylar notch and did not differ between sexes, while the border forming the DB was located further distally from the isthmus, between the middle and distal femoral segments. The feasibility of IM-guide usage is highly subject to DB, which is a limiting factor of IM rod engagement into the isthmic portion. In straight DB patterns, conventional use of the IM-guide with sole calibration of valgus resection angle according to dSAD is reliable due to the acceptable intramedullary canal geometry and stable purchasement of the IM rod in the isthmus. However, in bowed DB patterns, an alternative strategy should be adopted to avoid rod derailment from the desired course. Deakin et al. [8] reported a wide distribution of femoral mechanical–anatomical angles (FMAAs) ranging from 2° to 9° in the osteoarthritic population. They concluded that to achieve appropriate coronal alignment in TKA, the use of a fixed valgus resection angle is not suitable for all patients, and it may be preferable to adjust the distal femoral cut according to individual preoperative FMAAs and coronal alignment. Although their results regarding FMA distribution are concordant with dSAD distribution in our study, considerations about DB was lacking in their research (Table 6). In the present study, nearly half of male femora (110/232) had a dSAD of 4°–5.9° and two-thirds of female femora (294/424) had a dSAD of 4°–6.9°. Notably, the ratio of straight DB was predominant in individuals with a dSAD of 5°–7.9° but the ratio went much lower in individuals with dSAD < 5° or > 8°. The strategy for accurate distal femoral resection may thus require differentiation. In patients with dSAD < 5° or > 8°, the feasibility of IM-guide application decreases according to increasing DB, and simply changing the valgus resection guide angle may be insufficient to achieve the intended distal femoral resection angle. Depending on our measurement, mean femur length in male and female was 432.3 mm and 410.6 mm, respectively. In femora with mean length of the distal 1/3 femur 144.2 mm and 136.9 mm, 1° DB makes 2.5 mm and 2.4 mm deviation of the IM rod entry from the intercondylar notch ceiling. The deviation increases to 5.0 mm and 4.7 mm in 2° DB. Mullaji et al. ^9^ reported that the degree of preoperative deformity influenced valgus correction angle (VCA), and selecting a fixed VCA may result in unacceptable planning errors. They proposed that the HKA angle constituted a ‘deformity’ which influenced VCA. Our results indicated that the HKA angle influenced bowing or dSAD in both male and female, and the DB and dSAD increased in patients with HKA angle > 10°. Therefore, considering dSAD in combination with DB is the main factor determining the feasibility of IM-guide and additional strategies to overcome anatomical differences according to the HKA angle. In the correlation analysis between the dSAD and DB, femora with DB <  ± 1° were mostly had dSAD 4°–8°. IM-guide with adjustable VCA will be reliable for these population (Figs. 5 and 6). For the femora with ± 1° < DB <  ± 2°, IM-guide with adjustable VCA will be applicable with IM rod entry mobilisation. In the femora with ± 2° < DB and dSAD out of the 4°–8° interval, computer-assisted surgery including navigation or robotic systems in patients will likely show more reproducible result than IM-guide because DB >  ± 2° in this population will significantly reduce the applicability of IM-guide regardless of strategic variations.

This study has some limitations. First, the study analysed plain radiographic images. As plain radiography is inferior to computed tomography for depicting three-dimensional femoral anatomy, we could not analyse the influence of sagittal plane bowing or axial rotation on coronal femoral bowing. Second, this study involved human measurements and may have been subject to measurement errors. Nevertheless, we evaluated both inter- and intra-rater reliability and verified a high reliability. Finally, this study focused on the analysis of existing anatomy and adopted a non-comparative, non-clinical design. However, we attempted to identify and explain as many clinical implications as possible by comprehensively characterising the anatomical parameters of the femur.

A grossly straight femur with straight distal part was the most common femoral bowing pattern and constituted one-third of the Far East Asian population. Among grossly bowed femoral patterns, the distal bowing pattern was a key factor limiting the applicability of IM-guided TKA for distal femoral resection. Distal femoral resection using the IM-guide can be most beneficial in patients with a dSAD of 4°–8° and DB less than ± 1° with additional valgus resection guide calibration. IM-guide is expected to be applicable until DB less than ± 2°. However, in patients with dSAD < 4° or > 8° and DB exceeding ± 2°, utilisation of CAS-TKA is recommended in consideration of the increasing distal bowing angle, which interrupts the reliable application of an IM rod.

## Data Availability

Not applicable.

## Abbreviations

DB: Distal femoral bowing
dSAD: Distal segmental axial difference
FMA: Femoral mechanical–anatomical angle
GB: Gross femoral bowing
ICC: Intraclass correlation coefficient
IM: Intramedullary
HKA: Hip–knee–ankle
Ll: Grossly laterally bowed femora with laterally bowed distal part
Ls: Grossly laterally bowed femora with straight distal part
Mm: Grossly medially bowed femur with medially bowed distal part
mLDFA: Mechanical lateral distal femoral angle
mSAD: Middle segmental axial difference
NSA: Neck–shaft angle
PB: Proximal femoral bowing
pSAD: Proximal segmental axial difference
Sm: Grossly straight femur with medially bowed distal part
Ss: Grossly straight femur with straight distal part
TKA: Total knee arthroplasty
VCA: Valgus correction angle
